# Predictive role of blood eosinophils in adult varicella patients

**DOI:** 10.1017/S095026882200111X

**Published:** 2022-06-21

**Authors:** Luxuan Yang, Wenyong Zhang, Xiujuan Shen, Jianguo Chang, Meiqin Liu

**Affiliations:** Department of Infection Disease, The Fifth People's Hospital of Suzhou, Suzhou, Jiangsu, China

**Keywords:** Adult varicella, eosinophils, prediction index

## Abstract

The study aimed to explore the relationship between eosinophils and the prognosis of varicella in adults. We retrospectively reviewed the medical records of patients who were hospitalised in The Fifth People's Hospital of Suzhou with a diagnosis of adult varicella during the period between 1 January 2012 and 31 December 2020. Of the 359 patients, 228 (63.51%) had eosinopenia. The proportion of patients with mild type disease was significantly lower in the eosinopenia group than that in the non-eosinopenia group (50.44% *vs.* 65.65%, *P* = 0.006). The proportion of the patients with common type disease was significantly higher in the eosinopenia group than that in the non-eosinopenia group (39.47% *vs.* 28.24%, *P* = 0.039). The proportion of the patients with severe type disease was higher in the eosinopenia group, although the difference did not reach statistical significance (10.09% *vs.* 6.11%, *P* = 0.243). The rates of high fever (47.81% *vs.* 32.82%, *P* = 0.008; relative risk (RR) 1.296, 95% confidence interval (CI) 1.091–1.540), headache (43.42% *vs.* 22.14%, *P* < 0.001; RR 1.415, 95% CI 1.233–1.623), anorexia (53.51% *vs.* 35.88%, *P* = 0.001; RR 1.367, 95% CI 1.129–1.655) and complications (82.89% *vs.* 64.12%, *P* < 0.001; RR 2.106, 95% CI 1.460–3.038) were also significantly higher in the eosinopenia group. Among the complications, the liver injury and skin infection were more serious in the eosinopenia group. The disease course was significantly longer in the eosinopenia group than that in the non-eosinopenia group (9.43 ± 1.89 days *vs.* 8.73 ± 1.25 days, *P* < 0.001). The improvement rate of liver injury in the recovery period was lower in the eosinopenia group than that in the non-eosinopenia group (35.38% *vs.* 50%, *P* = 0.012). The study found that adult varicella patients with eosinopenia had a more serious condition, a higher morbidity of complications and a slower recovery. Blood eosinophils can be used as a new predictor of the severity of adult varicella.

## Introduction

Varicella is an acute infectious disease caused by varicella-zoster virus (VZV), which is commonly seen in children and has a serious clinical course in adults [1]. However, epidemiological investigations in recent years [2] have indicated an annual increase in the incidence rate of adult varicella in China and found that adult varicella patients had more serious complications and higher mortality, which directly indicates that more than half of the deaths of varicella were in older students and adults. As a result, it is particularly important to judge the severity of adult varicella patients as soon as possible and to take targeted countermeasures to improve the prognosis.

Eosinophils were considered as protective factors in respiratory virus infections in recent studies [3–6]. It has been demonstrated that the eosinophil antiviral response depends on the inclusion and production of molecules with antiviral activity, including RNA enzymes and active nitrogen species, the participation of eosinophils in adaptive immunity because they can act as antigen-presenting cells and the eosinophil extracellular traps [6]. Early in 2009, eosinophils recruitment to the lung tissue in response to active virus infection and protection against subsequently acquired acute respiratory virus infection were observed in a paradigm with pneumonia virus of mice [7] and similar findings were emerging. Eosinophils were capable of neutralising virus, reduced virus infectivity and promoted barrier responses in the early phase of influenza A virus infection [8]. Eosinophils were antiviral against parainfluenza virus via the production of nitric oxide and by serving as a dead-end host for virus infection [9]. Recently, it has also been suggested as a biomarker of poor outcome from COVID-19 in clinical studies [10, 11]. Therefore, we supposed there may be a mechanism that the inflammatory reaction and myelosuppression lead to the inhibition of the formation of eosinophils in bone marrow and the peripheral metastasis of mature eosinophils at an early stage of VZV infection, and these factors weaken the antiviral response of the eosinophils and worsened the disease. Combined with our clinical findings that some seriously ill adult varicella patients had eosinopenia, it can be considered that eosinophils may be related to the severity of varicella, but this has not been proven by current research. Therefore, our study possibly provided a new research direction for the early assessment of the severity of adult varicella patients by analysing the correlation between eosinophils and the disease severity of adult varicella patients.

## Materials and methods

Between 1 January 2012 and 31 December 2020, adult varicella patients who were admitted to The Fifth People's Hospital of Suzhou were considered eligible for enrolment. The inclusion criteria were as follows: (1) aged ≥18 years, (2) confirmed to have varicella due to having typical clinical manifestations or positive VZV immunoglobulin M (IgM) antibody and DNA tests; (3) VZV viraemia at admission (still had a fever and an emerging rash) and (4) complete clinical data. The exclusion criteria were as follows: (1) patients who were administered drugs that affected the patient's blood cell counts during the past month; (2) patients who had a history of infections, trauma or surgery during the past month; (3) patients who were pregnant or lactating and (4) patients who had acute or chronic hepatitis or nephritis, tumours, diseases of the blood system or immune system or other serious organ diseases.

The data collected and studied from medical records included age, sex, the time of onset and recovery, the highest temperature and the symptoms and complications during the disease course. The data obtained from the blood test results were the serum leucocyte count (WBC, 10^9^/l), serum haemoglobin (HGB, g/l), serum platelet count (PLT, 10^9^/l), serum lymphocyte count (LY, 10^9^/l), serum monocyte count (MON, 10^9^/l), serum eosinophil count (EOS, 10^9^/l), alanine aminotransferase level (ALT, U/l), aspartate aminotransferase level (AST, U/l), creatine kinase level (CK, U/l), lactate dehydrogenase level (LDH, U/l), serum C-reactive protein level (CRP), serum procalcitonin level (PCT) and the proportions of the different lymphocyte types, including CD4, CD8, B lymphocytes and natural killer cells. The blood samples were collected in the early morning after admission and discharge. The neutrophil-to-lymphocyte ratio (NLR) was calculated based on the blood cell parameters. According to whether there was high fever (the highest temperature during the course exceeded 39.1) and the severity of complications, the diseases were divided into mild (without high fever and serious complications), common (with high fever and no or minor complications) and severe (with serious complications regardless of the high fever). The serious complications included severe liver damage, thrombocytopenic purpura, severe electrolyte disorders, idiopathic thrombocytopenic purpura, viral pneumonia, myocarditis, encephalitis, sepsis and death.

### Statistical analysis

The data were analysed using SPSS (IBM SPSS Statistics 22.0). The count data are represented by the case number (*n*). The quantitative variables are expressed as the median (interquartile range). Categorical variables are expressed as proportions and percentages. The Mann–Whitney *U* test and Kruskal–Wallis test were used to compare the medians of the binomial and ordinal variables, respectively. Multivariable log-binomial models were used to estimate the relative risk (RR) of symptoms and complications based on the different levels of eosinophils. *P* < 0.05 was considered to indicate a statistically significant difference.

## Results

### General conditions of adult varicella patients

Of the 359 adult varicella patients, 227 (63.23%) were male, and 132 (36.77%) were female. All patients who had available vaccination records denied having been vaccinated with the varicella vaccine. These patients were aged from 18 to 45 years old, with an average age of 30.26 ± 5.86 years (107 patients (29.81%), aged ≥18 to <28; 234 patients (65.18%), aged ≥28 to <38; 18 patients (5.01%) aged ≥38 to <48). Weakness (75.49%), cough (62.95%), anorexia (47.08%) and high fever (42.43%) were the most frequent symptoms in our study (Table 1).

**Table 1.** General conditions of adult varicella patients

Characteristics	Number (*n*)	Percentage
Sex		
Male	227	63.23
Female	132	36.77
Age (years)		
≥18 to <28	107	29.81
≥28 to <38	234	65.18
≥38 to <48	18	5.01
Symptoms		
Weakness	271	75.49
Cough	226	62.95
Anorexia	169	47.08
High fever	152	42.34
Headache	128	35.65
Abdominal discomfort	76	21.17
Chest discomfort	45	12.53

### Comparison of the severity between the eosinopenia group and the non-eosinopenia group

Since the normal range of the serum eosinophil counts in this study was (0.02–0.52) × 10^9^/l and there were only two cases with mildly elevated eosinophils (one was 600/μl and the other was 1300/μl), the groups were divided according to whether the eosinophil count was less than 20/μl. Among the 359 adult varicella patients, 228 (63.51%) had eosinopenia, and 131 (36.49%) had normal eosinophil counts. The proportion of patients with the mild type was significantly lower in the eosinopenia group than in the non-eosinopenia group (50.44% *vs.* 65.65%, *P* = 0.006). The proportion of patients with the common type was significantly higher in the eosinopenia group than that in the non-eosinopenia group (39.47% *vs.* 28.24%, *P* = 0.039). The proportion of patients with the severe type was higher in the eosinopenia group, although the difference did not reach statistical significance (10.09% *vs.* 6.11%, *P* = 0.243) (Table 2).

**Table 2.** Severity between the eosinopenia group and the non-eosinopenia group

	Eosinopenia group	Non-eosinopenia group	*P*-value
Mild type	115 (50.44%)	86 (65.65%)	0.006
Common type	90 (39.47%)	37 (28.24%)	0.039
Severe type	23 (10.09%)	8 (6.11%)	0.243

Mild type: without high fever and serious complications. Common type: with high fever and no or minor complications. Severe type: with serious complications regardless of the high fever.

### Comparison of the characteristics between the eosinopenia group and the non-eosinopenia group

The sex and age differences between the two groups were not statistically significant. There was no difference between the groups regarding weakness and cough, which were two of the most frequent symptoms. The rate of high fever (47.81% *vs.* 32.82%, *P* = 0.008; RR 1.296, 95% confidence interval (CI) 1.091–1.540), headache (43.42% *vs.* 22.14%, *P* < 0.001; RR 1.415, 95% CI 1.233–1.623), anorexia (53.51% *vs.* 35.88%, *P* = 0.001; RR 1.367, 95% CI 1.129–1.655) and complications (82.89% *vs.* 64.12%, *P* < 0.001; RR 2.106, 95% CI 1.460–3.038) were higher in the eosinopenia group (Tables 3 and 4). There was no significant difference in the degree of liver damage between the two groups, but the median ALT and AST levels in the eosinopenia group were significantly higher. Skin infection refers to a bacterial infection secondary to a broken skin rash that is complicated with serious manifestations, such as a deep abscess or sepsis. None of the patients in the non-eosinopenia group had deep abscesses or sepsis, and there were six cases of skin infections in the eosinopenia group, of which four patients had deep abscesses and two patients had sepsis (Table 5).

**Table 3.** Sex, age, uncomfortable symptoms and complications between the eosinopenia group and the non-eosinopenia group

	Eosinopenia group	Non-eosinopenia group	*P*-value
Male	140 (61.40%)	87 (66.41%)	0.428
Female	88 (38.60%)	44 (33.59%)	
Age (years)	30.36 ± 5.96	30.08 ± 5.76	0.938
Symptoms			
Weakness	178 (78.07%)	93 (70.99%)	0.161
Cough	142 (62.28%)	84 (64.12%)	0.820
Anorexia	122 (53.51%)	47 (35.88%)	0.001
High fever	109 (47.81%)	43 (32.82%)	0.008
Headache	99 (43.42%)	29 (22.14%)	0.000
Abdominal discomfort	47 (20.61%)	29 (22.14%)	0.789
Chest discomfort	34 (14.91%)	11 (8.40%)	0.097
Complications			
Total	189 (82.89%)	84 (64.12%)	0.000
Liver damage	130 (57.02%)	60 (45.80%)	0.048
Skin infection	39 (17.11%)	11 (8.40%)	0.026
Bacterial pneumonia	15 (6.58%)	11 (8.40%)	0.532
Electrolyte disorder	14 (6.14%)	9 (6.87%)	0.825
Idiopathic thrombocytopenic purpura	14 (6.14%)	6 (4.58%)	0.637
Viral pneumonia	2 (0.88%)	2 (1.53%)	–
Myocarditis	2 (0.88%)	0 (0%)	–
Encephalitis	1 (0.44%)	0 (0%)	–

**Table 4.** Associations between eosinopenia and high fever, headache, anorexia, complications

	Exposed/unexposed	RR (95% CI)	Adjusted RR (95% CI)
High fever	109/43	1.287 (1.083–1.529)	1.296 (1.091–1.540)
Headache	99/29	1.376 (1.189–1.592)	1.415 (1.233–1.623)
Anorexia	122/47	1.379 (1.141–1.667)	1.367 (1.129–1.655)
Complications	189/84	2.097 (1.454–3.025)	2.106 (1.460–3.038)

RR, relative risk; adjusted RR, adjusted for sex and age.

**Table 5.** Liver damage and skin infection between the eosinopenia group and the non-eosinopenia group

	Eosinopenia group	Non-eosinopenia group	*P*-value
Degree of liver damage			
1 ULN < ALT < 2 ULN	91 (70%)	44 (73.33%)	0.732
2 ULN ≤ ALT ≤ 10 ULN	37 (28.46%)	16 (26.67%)	0.863
ALT > 10 ULN	2 (1.54%)	0 (0%)	–
ALT (U/l)	41 (29, 62)	35 (23, 65)	0.042
AST (U/l)	37 (27, 49)	27 (22, 41)	0.000
Severity of skin infection			
No deep abscess or sepsis	33 (84.62%)	11 (100%)	–
With deep abscess or sepsis	6 (15.38%)	0 (0%)	

### Comparison of the prognosis between the eosinopenia group and the non-eosinopenia group

The duration of disease in the eosinopenia group (9.54 ± 1.86 days) was longer than that in the non-eosinopenia group (8.73 ±1.25 days) (*P* < 0.001), and the number of patients with liver damage improvement (46 (35.38%)) in the eosinopenia group was less than that (33 (50.00%)) in the non-eosinopenia group (*P* = 0.012). The liver function was reviewed in the patients: 16 patients in the eosinopenia group had normal liver function and eight patients (50%) in the eosinopenia group had new liver damage. However, one patient (33.33%) in the non-eosinopenia group had new liver damage (Table 6).

**Table 6.** Prognosis between the eosinopenia group and the non-eosinopenia group

	Eosinopenia group	Non-eosinopenia group	*P*-value
Duration of disease (days)	9.54 ± 1.86	8.73 ± 1.25	0.000
Patients with liver damage improved	46 (35.38%)	33 (50%)	0.012
Patients with liver damage advanced	35 (26.92%)	12 (20%)	0.469
Patients with liver damage emerged	8 (50%)	1 (33.33%)	–
Patients with abandoning treatment	1 (0.44%)	0 (0%)	–

### Analysis of the infection indexes and lymphocyte subsets between the eosinopenia group and the non-eosinopenia group

After comparing infection indexes and lymphocyte subsets, it was found that the NLR level in the eosinopenia group was higher than that in the non-eosinopenia group (1.38 *vs.* 1.18, *P* = 0.011), and the proportion of B lymphocytes was lower than that in the non-eosinopenia group (5.09% *vs.* 8.00%, *P* = 0.001) (Table 7).

**Table 7.** Infection indexes and lymphocyte subsets between the eosinopenia group and the non-eosinopenia group

	Eosinopenia group	Non-eosinopenia group	*P*-value
CRP (mg/l)	17.5 (9.4–30.0)	18.0 (7.3–30.8)	0.703
PCT (ng/ml)	0 (0–0.29)	0 (0–0.25)	0.168
NLR	1.38 (0.92–2.38)	1.18 (0.87–1.68)	0.011
CD4^+^ T cell (%)	29.74 (22.00–38.56)	30.00 (24.37–37.02)	0.860
CD8^+^ T cell (%)	48.10 (33.89–60.00)	46.30 (35.25–60.30)	0.881
B cell (%)	5.09 (3.63–8.01)	8.00 (5.00–11.00)	0.001
NK cell (%)	11.31 (8.81–15.96)	12.63 (9.00–16.87)	0.299

## Discussion

Normally, we tend to focus more on immunocompromised people because severe varicella mostly occurs in children and immunodeficient people [12]. However, in recent years, it has been reported that adult varicella patients with normal immune function have serious complications, such as acute respiratory distress, liver failure and cerebral venous sinus thrombosis [13–16]. Moreover, as adults typically do not voluntarily vaccinate against varicella, varicella infections in adults are likely undervalued and understudied. To fill the gap in knowledge, this study was designed to establish methodologies to determine the severity of adult varicella using peripheral blood eosinophils as a correlate for severity.

Based on the clinical data of adult varicella, this study found that patients with eosinopenia had more serious disease, had a higher morbidity of complications and had a longer recovery time, which was a manifestation of persistent viraemia. It has been demonstrated that eosinophils have many important biological functions, including immune regulation, tissue remodelling and repair, maintaining homoeostasis, exhibiting anti-inflammatory and antitumor activity [17] and initiating antiviral responses against some viral infections [7–11]. Therefore, it can be inferred that eosinophils can reduce virus replication by inducing CD8T cell proliferation, activation and effector functions [18], therefore, eosinopenia can delay VZV clearance and cause persistent inflammation, which causes serious complications and poor prognosis. Notably, the antiviral response of eosinophils often appears in ssRNA viral infections [7–11], and VZV is a DNA virus. Whether the findings of this study expand the antiviral scope of eosinophils or indicate that other immune mechanisms are involved remains to be further studied.

It has been demonstrated that eosinophils act as antigen-presenting cells in response to viral antigens and are capable of inducing cytokine secretion by T cells [5,6]. However, a significant decrease in the proportion of B cells in the eosinopenia group and no significant difference in the number of T cells between the two groups were found in this study. The disease severity of a primary VZV infection is increased in individuals with compromised invariant natural killer T cells immune responses [19], and iNKT cells can influence the immediate responses by innate-like B cells, which are critical for initiating the early responses against systemic pathogens [20]. Meanwhile, there was a correlation between eosinophils and B cells in this study. Recent studies [21–23] have found that there is a correlation between B cells and viral infections because B cells can secrete interleukin-10 (IL-10), IL-35 and transforming growth factor-*β* and play a negative immunomodulatory role. This reminds us that we should pay attention to the role of innate immunity in VZV infection, as this will be helpful in judging the severity of the disease and selecting appropriate treatment.

The NLR is widely used across almost all medical disciplines as a reliable marker of the immune response to various infectious and non-infectious stimuli [24]. However, the application of the NLR in patients with viral infection is mostly limited to the prognostication of severe acute respiratory syndrome coronavirus 2 (SARS-CoV-2) infection and associated patient outcomes [25–27]. Considering eosinophils attenuate viral infectivity through production of nitric oxide [9] and a high NLR is associated with excessive levels of reactive oxygen species [28], we speculated that eosinopenia and the increase of NLR were associated with a decrease in antioxidant defences, which can promote inflammation and oxidative damage during infection. If this is true, antioxidants could be considered for severe patients.

The proportion of males was significantly higher than that of females in all of the groups, but this difference disappeared after the patients were divided into the eosinopenia group and the non-eosinopenia group. On the one hand, pregnant women were excluded from this study in order to focus on immunocompetent adults, which may have led to selection bias. On the other hand, there are sex differences in the immune response. Some critical immune-related genes can escape from X chromosome inactivation in some cells, which leads to higher gross expression of some of the immune-related genes in females, and females generally mount more pronounced cytokine responses in viral infections [29]. Therefore, females infected with VZV may not develop disease or may only have a mild disease as a result of a stronger immune response that can clear the virus, and these patients often choose to not seek medical advice. This would be a great explanation for the lower morbidity of females; however, in this study, there was no sex difference between the two groups, revealing that male sex may be a risk factor for adult varicella.

In this study, all cases of deep abscesses and sepsis secondary to skin infections appeared in the eosinopenia group, which was consistent with previous research that demonstrated eosinophils may be a marker during bacterial infections [3, 4]. Thus, we can make better diagnoses and reduce antibiotic abuse in adult varicella patients with eosinophils. At the same time, our study found that the proportion of liver damage in the population with low eosinophils was higher, the disease severity was more serious and the disease recovery was slower. A study [30] has suggested that eosinophils accumulate in the injured liver when the liver injury is caused by immune reactions and that eosinophils secrete IL-4 in local tissues, which can induce the proliferation of resting hepatocytes and regulate liver regeneration. It is speculated that a low eosinophil count may affect the regeneration of liver cells. However, it is not clear whether eosinophils are consistent in the serum and tissues, and this study failed to collect enough eosinophil values in the patients during the whole course of disease. In a follow-up study, eosinophil values at multiple time points will be collected, and eosinophils in the liver will be studied to further determine the correlation.

In terms of vaccine safety, a study pointed out there is considerable concern about whether SARS-CoV-2 exposure post-vaccination would cause eosinophil-associated lung pathology through immunopotentiation [5]. Considering that the study only emphasised the exclusion of eosinophil-associated disease and varicella vaccine is overall safe in long-term review [31], the safety of varicella vaccination can be ensured. We found that all the patients who had available vaccination records denied vaccination with the varicella vaccine in this study. On the one hand, varicella vaccines are not widely available in their childhood; on the other hand, the public often believes that varicella is a childhood epidemic, which leads to the neglect of adult varicella. Therefore, it is necessary to strengthen the public awareness of this disease, increase the attention regarding the importance of adult varicella and improve the varicella vaccination rate in adults.

Generally, we can improve the adult varicella prognosis by clearing VZV and reducing inflammatory reactions as soon as possible. However, to date, there are no effective anti-VZV drugs, the immune inhibitors that can reduce inflammation may cause internal disseminated varicella, and the use of Ig is costly. Other than observing the patient's eosinophil levels and giving the patient timely symptomatic treatment, regrettably, there seem to be few effective methods for this disease. If the related mechanisms of eosinophils can be further elucidated and if the disease can be treated in the early stage, the severity of the disease can be effectively improved and a favourable prognosis will be obtained.

## Conclusion

The importance of varicella in adults has been seriously underestimated, and there is no research on how to predict the severity of varicella disease, which needs to be filled in the corresponding gaps. In this study, a retrospective analysis was conducted based on the research and clinical experience of other viruses, and eosinophils were found to be a simple and effective predictor, but the specific cut-off value and its internal mechanism need to be further studied. Meanwhile, considering the anti-inflammatory and virus-clearing functions of eosinophils, whether eosinophils can be used as therapeutic targets can be the next research direction.

## Data Availability

The datasets used and/or analysed in the current study are available from the corresponding author on reasonable request.
